# Comparison of surgeon-performed ultrasound-guided fine needle aspiration cytology with histopathological diagnosis of thyroid nodules

**DOI:** 10.12669/pjms.35.4.537

**Published:** 2019

**Authors:** Muhammad Ayoob Jat

**Affiliations:** Dr. Muhammad Ayoob Jat, Assistant Professor Surgery, Department of Surgery, College of Medicine, Northern Border University, Arar, KSA

**Keywords:** Fine needle aspiration cytology, Histopathology, Solitary thyroid nodule

## Abstract

**Objective:**

To assess the Solitary thyroid nodules by surgeon- performed ultrasound-guided FNAC and evaluate with the histopathological findings.

**Methods:**

This study includes 100 Consecutive patients of a solitary thyroid nodule which were presented to the Outpatients Department of Surgery during the period of two years from September 2016 to August 2018. Exclusion criteria were patients with extra-thyroid swelling, diffuse goiter and multinodular goiter. All patients with a solitary thyroid nodule underwent Surgeon –performed ultrasound-guided FNAC in the department of Radiology. After thyroid surgery, thyroid specimens were sent for histopathology and evaluate with FNAC findings.

**Results:**

The study included hundred patients with solitary thyroid nodule, 75(75%) female and 25 (25%) male with a ratio of F 3:1M. The age of the patients ranged from 15-75 years with a mean age of 35 years. The result of 100 cases of Surgeon –performed Ultrasound –guide FNAC of a solitary thyroid nodule were inconclusive in 10 cases (10%), Non-neoplastic in 60 cases (60%) and Neoplastic lesions in 30 cases (30%). After evaluation of findings from FNAC and histopathology, four cases with benign FNAC (adenomatous/colloid Goiter) turnout as neoplastic (papillary carcinoma) on histopathology and six cases with neoplastic FNAC (papillary carcinoma), just two cases turnout as benign (nodular colloid goiter with cystic degeneration) on histopathology. In present study Surgeon – performed Us FNAC has found to be 87.5% sensitive, 95.3% specific and 92.0% diagnostic accuracy.

**Conclusion:**

Surgeon – performed Ultrasound-guided FNAC is a safe, simple and accurate technique in the diagnosis of solitary thyroid nodule.

## INTRODUCTION

Solitary thyroid nodule is a common surgical problem presents in surgical clinic at outpatients department. .Solitary thyroid nodule is defined clinically as a palpably discrete swelling within an otherwise apparently normal gland, is usually a benign lesion. A different diagnostic tools like ultrasound, thyroid nuclear scan, and fine needle aspiration cytology (FNAC) are available to the clinician for evaluation of thyroid nodule but FNAC is considered the gold standard diagnostic tool in the evaluation of a thyroid nodule, The prevalence of thyroid nodules has been estimated to be as high as 64%, with the incidence of malignancy ranging from 5 to 10%, depending on the population under study.^1^

Fine needle aspiration cytology (FNAC) is a rapid, minimally invasive, and cost-effective first-line an invaluable tool for Us-FNAC the establishing the diagnosis of thyroid nodules.^2^ Ultrasound–guided FNAC has a higher diagnostic accuracy than conventional (palpation-guided) FNAC.^3^ In current clinical practice, it is important for a surgeon to be capable of performing Us-FNAC for a broad range of head and neck sites including the thyroid, salivary glands, and lymph nodes. However, most studies of surgeon-performed USFNAC have focused on thyroid nodules.^4^ On review of literature there is evidence that ultrasound -guided surgeon-performed FNAC carries a number of benefits, including reduction of multiple physician visits, assessment by a physician familiar with neck anatomy, as well as potentially decreasing wait times to surgery.^5^

The purpose of this study was to evaluate the Solitary thyroid nodules by surgeon-performed ultrasound-guided FNAC and compare with the histopathological diagnosis. Also to determine the sensitivity, specificity and accuracy of Surgeon-performed Ultrasound-guided FNAC.

## METHODS

This cross-sectional prospective study was conducted for the period of two years from Sept 2016- August 2018 in the Department of surgery, at central hospital, Arar, KSA. *During this study period we accepted all patients came in OPD with clinically diagnosed as a solitary thyroid nodule having no hyper or hypothyroidism, irrespective of age and sex (inclusion criteria). Exclusion criteria were patients presenting with extra-thyroid neck swelling*. This study is not applicable in patients having toxic or non- toxic diffuse or multinodular goiter. A total of 100 patients with solitary thyroid nodule were presented in Surgical Clinic during study period. A detailed Clinical history and thorough physical examination was carried out and recorded on clinical proforma. Routine blood chemistry, Ultrasonography of Neck, thyroid function tests in all cases but radioiodine scan, computed tomography in selected cases. A well-informed consent was taken from all patients. The technique, risks, benefits, and associated complications of the procedure were explained to all patients.

Ultrasound-guided FNAC was performed by surgeon in all cases of solitary thyroid nodule in the Department of Radiology. The patient was placed in a supine position with the neck extended and the right-handed surgeon at the right side of the patient. A G.E. LOGIC – E9 Ultrasound system with a high-frequency linear 6–15 MHz transducer was used. The Us-FNAC procedure was performed using a non-aspiration capillary technique with two passes of a 1.5-inch 21-gauge needle. The specimen was deposited onto a glass slide and smeared with another slide. The sample was immediately fixed to avoid desiccation artifacts. The collected material is placed on four glass slides, smeared, and fixed in 95% ethyl alcohol for about 30 minutes. All the slides were stained with Papanicolaou stain. Specimens were reviewed for cellular adequacy onsite by the cytopathologist at the time of the procedure. The smear was considered adequate if there were at least five groups of well-visualized follicular cells, each group containing ten or more cells. In this study no complications associated with US-FNAC were reported.

About 75 patients subsequently underwent thyroid surgery and thyroid specimens were sent for histopathology then finally FNAC findings were compared with the histological findings to assess the sensitivity, specificity, positive predictive value, negative predictive value and overall diagnostic accuracy of Surgeon-performed Ultrasound –guided Fine needle aspiration cytology.

## RESULTS

A total of 100 patients with solitary thyroid nodule were presented in Surgical Clinic 75(75%) female and 25 (25%) male with a ratio of F 3:1M during study period. The age of the patients ranged from 15-75 years with a mean age of 35 years. The FNAC findings in this series for distribution of solitary thyroid lesion were: inconclusive FNAC in 10 cases (10%), Non-neoplastic in 60 cases (60%) and Neoplastic in 30 cases (30%). As shown in Table-I.

**Table I:** Distribution of FNAC Findings in the Solitary Thyroid Nodules (n=100).

	Type of lesion	No	%
Non-diagnostic / Inconclusive		10	10%
Non- neoplastic		60	
	Hyperplastic nodule	12	20%
	Adenomatous goiter	25	41.6%
	Hemorrhagic colloid nodule	15	25%
	Hashimoto thyroiditis	5	8.3%
	Colloid cyst	3	5%
Neoplastic		30	
	Follicular Neoplastic	22	73.4%
	Hurthle cell adenoma	2	6.6%
	Papillary Carcinoma	6	20%

In this study 75 cases were operated for thyroid surgery so their histopathological specimen available for comparison between FNAC and histopathological findings. Out of 45 non neoplastic lesions on FNAC, four lesions were turnout as neoplastic lesion on histopathology so accuracy of FNAC diagnosis was 91.1%, while in neoplastic group, out of 30 patients, two cases was turnout as Non neoplastic lesion on histopathology so the accuracy rate was 93.3% as showed in Table-II.

**Table II:** Cytohistopathological correlation of thyroid lesions (n=75).

S. No	FNAC Diagnosis	No = 100	Thyroid Specimens N=75	Match	Mismatch	Histopathological diagnosis
1	Non-diagnostic / Inconclusive	10	-	-	-	NA
2	Adenomatous /colloid Goiter	40	40	36	4	36 Colloid Goiter 4 Cystic Papillary carcinoma
3	Hyperplastic nodules	12	--	--	--	NA
4	Hashimoto thyroiditis	5	5	5	---	5 Hashimoto thyroiditis
5	Colloid cyst	3	--	--	---	NA
6	Hurthle cell neoplasm	2	2	2	---	2 Hurthle cell adenoma
8	Follicular Neoplasm	22	22	22		20 Follicular adenoma 2 Follicular carcinoma
9	Malignant Papillary carcinoma	6	6	4	2	4 Papillary carcinoma 2 Nodular colloid goiter with cystic degeneration

	Total	100	75	69	06	

### Abbreviations

**FN:** False negative; **FP:** False positive, **TN:** True negative, **TP:** True positive.

### Statistical Analysis of Thyroid Nodules

Statistical analysis was done using MS Excel 2010. In this study the sensitivity, specificity, positive predictive value, negative predictive value and overall diagnostic accuracy of Fine needle aspiration cytology were calculated by correlating the results of cytology with histopathology by using Galen and Gambino method with following formulas. Table-III.

**Table III:** Analysis of FNAC and Histopthological diagnosis of Solitary thyroid nodules (n=75).

		Histopatho	Logy	Total	Discordant cases
		Benign	Malignant		
CYTOLOGY	Benign	41 (TN)	4 (FN)	45	4 (FN)
	Malignant	2 (FP)	28 (TP)	30	2 (FP)

TOTAL		43	32	75

1. Sensitivity = TP /TP+FN x100 --------------- = 87.5%
2. Specificity = TN /TN+FP x 100 --------------- = 95.3%
3. Positive predictive value = TP /TP + FP x 100--- = 93.3%
4. Negative predictive value = TN / TN+FN x100 = 91.1%
5. Accuracy = TP +TN /TP+TN+FP+FN x100 = 92.1%

## DISCUSSION

Surgeon-performed Ultrasound-guided FNAC has gained popularity in the recent literature, as a method to further decrease surgical wait-time as well as increase patient convenience and satisfaction due to a decreased number of specialist referrals^6^,^7^ Ultrasound- guided FNAC improves the cytological diagnostic accuracy, compared to palpation-guided FNAC.^8^ In present study the accuracy of Surgeon –performed -Us-FNAC is 92% but in some studies accuracy up to 97% in the preoperative diagnosis of various thyroid lesions.^9^,^10^ The adequacy rate of FNAC is dependent on the availability of a cytopathologist for immediate specimen assessment.^11^,^12^ Surgeon-performed ultrasound (SPUs) has become an extension of the physical examination in the evaluation of patients with thyroid nodules.^13^ In general, histological examination must be done to distinguish between follicular carcinoma and benign follicular adenoma as invasion through the tumor capsule or vascular invasion is evidence of carcinoma. International guidelines recommend that repeat aspiration under Ultrasound- guidance to augment the accuracy in 15–25% of thyroid nodules in which FNA yields inadequate diagnostic material.^14^

This study includes hundred patients with solitary thyroid nodule, 75(75%) female and 25 (25%) male with a ratio of F 3:1M. The age of patient ranged from 15- 75 years with a mean age of 34 years .In study by Dr. Shemy et al.15 out of 50 cases of thyroid nodules 41 were female and 9 were male with a ratio of (F: M – 5 :1), common in the age group of 30- 39 years. The findings of Surgeon-performed Us- FNAC of solitary thyroid nodule shows 10 cases (10%) were inconclusive/ Non-diagnostic, 60 cases (60%) Non-neoplastic (40 adenomatous/colloid goiters, 12 hyperplastic nodules, five Hashimoto thyroiditis, three colloid cyst) and 30 cases (30%) Neoplastic lesions (follicular neoplasm 22(73.4%), Hurthle cell adenoma 2 (6.6%), papillary carcinoma 6 (20.0%).This study shows that non-diagnostic rate of Usg- FNAC was 10% (10 /100) similar result seen in study by Oktay I et al 10% (47 of 470)16, but 5.6% in study by Bohacek L et al.17 A major factor associated with inadequate US-FNAC results is cystic changes within the tumor and another factor is presence of rim calcification.18,19 All 10 patients with inconclusive FNAC reports (10%) underwent repeat FNAC, which was turnout as benign in 8 cases (80%) and non-diagnostic in two cases. All these Patients were followed with serial Ultrasound and remain stable.

The present study shows 45 cases with benign FNAC, concordance in 41 cases (True Negative) and discordance in four cases (False Negative) turnout as papillary carcinoma and 30 case with malignant FNAC, concordance in 28 cases (True Positive) two cases turnout as nodular colloid goiter with cystic degeneration (False positive). The fine needle cytology aspiration in four cases (False negative) diagnosed as nodular goiter with cystic degeneration consisted of straw colored fluid with foamy macrophages and abundant colloid were subsequently confirmed on histopathology as papillary carcinoma. Cystic papillary carcinoma is the commonest cause of false negative reports 17. In this series six cases of papillary carcinoma on cytology, four were confirmed on histopathology, remaining two were nodular colloid goiter with cystic degeneration. Hence diagnostic accuracy of fine needle aspiration for papillary carcinoma was 66.6%.

This study shows that the sensitivity, specificity and diagnostic accuracy of Surgeon –performed Ultrasound –guided FNAC for solitary thyroid nodules were 87.5%, 95.3%, and 92.0% respectively. In study by Dr. Yagesh P et al.,^20^ the sensitivity is 70% specificity is 94.7% and diagnostic accuracy is 86.2%. In same study Out of total 90 cases of thyroid nodules FNAC Only 29 cases was operated and available for correlation with histological findings, in which 21 were diagnosed as benign and 8 as malignant on cytology, whereas 19 cases were diagnosed as benign and 10 as malignant on histopathology. There were 3 (false negative) and 1 (false positive) cases. In various studies by Amatya B et al.,^21^ Rathod GB et al.22 and Musani MA et al.,^23^ found incidence of false negative cases 5.4%, 13.6% and 4.7% respectively. False negative diagnosis in the cases with follicular patterns is one of the major FNAC limitations. FNAC cannot distinguish Carcinoma and follicular neoplasms thus excision is must for an accurate and definite diagnosis.

## CONCLUSION

Surgeon – performed US-guided FNAC is a safe, simple and accurate technique in the diagnosis of solitary thyroid nodule. A team work between Surgeon, cytopathologist, and radiologist maximizes the diagnostic accuracy of FNAC. Repeated Ultrasound- guided FNAC has high diagnostic value in thyroid nodule where FNAC was insufficient or inconclusive.
